# Effectiveness of Whey Protein Supplementation in Weight Loss Interventions for Patients with Obesity: A Systematic Review

**DOI:** 10.3390/nu18040695

**Published:** 2026-02-21

**Authors:** Juan José López-Gómez, Beatriz Ramos-Bachiller, Daniel Rico-Bargues, Daniel A. De Luis-Román

**Affiliations:** 1 Servicio de Endocrinología y Nutrición, Hospital Clínico Universitario de Valladolid, Avenida Ramón y Cajal, 3, 47003 Valladolid, Spain; 2 Health Research Institute of Valladolid (IBioVALL), Calle Rondilla de Santa Teresa s/n, 47010 Valladolid, Spain; 3 Centro de Investigación de Endocrinología y Nutrición, Fac Medicina, Universidad de Valladolid, Avenida Ramón y Cajal, nº7, 47005 Valladolid, Spain; 4 Centro de Investigación Biomedica en Red de la Obesidad y Nutrición (CIBEROBN), Instituto de Salud Carlos III, Avenida Monforte de Lemos, 3-5, Pabellón 11, Planta 0, 28029 Madrid, Spain

**Keywords:** obesity, sarcopenic obesity, weight loss, bariatric surgery, obesity drugs, diet, exercise

## Abstract

**Background**: Obesity is traditionally defined by excess fat mass; however, the preservation of fat-free mass (FFM), particularly skeletal muscle, has gained increasing relevance due to its metabolic, endocrine, and functional roles. Weight loss interventions, including hypocaloric diets, pharmacological treatments, and bariatric surgery, are frequently associated with unintended loss of skeletal mass, increasing the risk of sarcopenic obesity and related complications. **Objective**: This study aimed to systematically evaluate the effectiveness of whey protein supplementation in preserving fat-free mass and muscle-related outcomes in adults with obesity undergoing weight loss interventions. **Methods**: A systematic review was conducted in accordance with PRISMA guidelines. Randomized controlled trials published in English were identified through searches of PubMed/MEDLINE, CENTRAL, Embase, Scopus, ClinicalTrials.gov, and WHO ICTRP, searched up to September 2025. Eligible studies included adults (>18 years) with obesity receiving whey protein supplementation as part of a hypocaloric diet, compared with placebo or standard interventions. Primary outcomes were changes in fat-free mass assessed by validated methods (DXA, BIA, MRI), while secondary outcomes included body weight, fat mass, metabolic parameters, adherence, and safety. Risk of bias was assessed using the Cochrane RoB 2.0 tool, and certainty of evidence was evaluated with GRADE. The abstract was registered in PROSPERO with code CRD420251069996. There was no funding and no conflicts of interest. **Results**: Fourteen randomized controlled trials were included. Whey protein supplementation generally supported the maintenance or modest improvement of fat-free mass, particularly when combined with resistance exercise or anabolic-enriched formulations such as leucine or vitamin D. Several trials, however, reported neutral effects, especially in the absence of structured physical activity. Overall, effect estimates ranged from small gains to null or uncertain differences, and the certainty of evidence was frequently downgraded due to limited sample sizes, wide confidence intervals, heterogeneity across interventions and assessment methods, short follow-up periods, and methodological limitations including open-label designs and inconsistent adherence monitoring. **Conclusions**: Whey protein supplementation may support fat-free mass preservation during weight loss in adults with obesity, particularly as part of a multimodal intervention. Further high-quality trials are needed to define optimal dosing strategies and target populations.

## 1. Introduction

Obesity is a highly prevalent disease that affects 13% of adults, with more than 890 million over the body mass index 30 kg/m^2^ threshold in 2021 [1]. Globally, the prevalence and incidence of this disease continue to rise, with marked, age, sex and racial differences. The main treatments for obesity are intensive lifestyle interventions (behavioral modification, nutrition, and physical activity), pharmacotherapy, and metabolic/bariatric procedures. Although all interventions are designed to reduce fat mass, they may also have detrimental effects on lean mass, an impact that becomes more pronounced as the intensity of therapy increases [2].

Obesity is characterized by an increase in fat mass, but the proportion of fat-free mass, which includes muscle mass, is now also considered important in the nutritional assessment of patients. Muscle performs endocrine, immunological, and neurological functions, in addition to providing structural support to the skeleton, through the release of myokines [3]. Patients with obesity are at risk of a reduction in both the quantity and function of muscle mass, defined as sarcopenia [4]. Among the risk factors that predispose patients with obesity to sarcopenia are insulin resistance and chronic inflammation, which promote gluconeogenesis and the breakdown of muscle proteins, as well as inadequate protein intake and intestinal dysbiosis [5]. Bariatric surgery and GLP-1 analogs may act as triggers for the loss of fat-free mass if dietary and physical activity recommendations are not properly addressed [5,6].

Sarcopenia and obesity frequently appear together in a condition termed sarcopenic obesity, characterized by the gradual decline of skeletal muscle mass and function alongside an increase in adiposity. This combination is often fueled by lifestyle factors, particularly physical inactivity and poor nutrition, that contribute to metabolic disturbances and chronic low-grade inflammation [7]. From a dietary standpoint, insufficient energy and protein intake is a key cause of secondary sarcopenia. Although ensuring adequate protein consumption (at least 1.0 g/kg/day in older individuals with overweight or obesity) helps protect against the loss of skeletal muscle mass, its benefits are markedly amplified when paired with regular physical activity; resistance exercise especially provides a potent stimulus for muscle protein synthesis and can substantially increase skeletal muscle mass and strength combined with protein supplementation. For these reason, effective management of sarcopenia requires nutritional interventions with structured exercise programs to enhance overall functional capacity [7].

Sarcopenic obesity carries a higher risk of cardiovascular disease, falls, fractures, metabolic complications, and mortality than obesity alone [3]. Moreover, diabetes mellitus constitutes an additional risk factor for the development of sarcopenia, and the relationship appears to be bidirectional, which in patients with obesity perpetuates a vicious cycle, further increasing cardiovascular risk and its associated complications [8]. Advanced age predisposes individuals to the accumulation of visceral and intra-abdominal fat, promoting fat infiltration of skeletal muscle and leading to a loss of strength and functional capacity [9]. Given the progressive aging of the population and the increasing prevalence of obesity, the number of people at risk of sarcopenia is steadily rising, making the study of its prevention and management essential.

Key pillars in preventing the loss of skeletal muscle mass in patients with obesity include adequate protein intake and the performance of strength–resistance exercises. Protein deficiency is the most common nutritional deficiency among patients with obesity [7]. Protein intake is often reduced in individuals with excess weight, with a predominance of carbohydrate and saturated fat consumption [10]. Protein is the most satiating macronutrient, with a linear relationship between the sensation of satiety and the amount of protein consumed [11], whereas fats and carbohydrates have a much more limited satiating capacity. This partly explains dietary approaches focused on increasing protein intake in these patients [12].

High-protein diets have been shown to achieve greater satiety, increased thermogenesis—with the resulting loss of fat mass—and better preservation of fat-free mass compared with normoproteic diets, especially when combined with physical activity [13,14]. However, due to heterogeneity in study samples, the amount and type of protein used, and inconsistent reporting of accompanying physical activity, it is difficult to establish a standard recommendation regarding the optimal amount and type of protein for these patients.

The use of protein supplementation in patients with obesity has also been previously studied, although with contradictory results. While some studies report that protein supplementation combined with a hypocaloric diet attenuates the loss of fat-free mass—particularly in cases of rapid and significant weight loss, such as after bariatric surgery [12,15,16]—other studies find a neutral or even detrimental effect on the preservation of fat-free mass [17]. The use of short peptides, leucine, or hydroxymethylbutyrate [18] may help promote beneficial effects on muscle, as may the use of probiotics [19], which have been associated with favorable effects on gut microbiota and the gut–brain axis involved in satiety and the release of intestinal peptides. However, studies evaluating the effects of protein supplementation are similarly heterogeneous, making universal recommendations difficult.

The purpose of this review is to synthesize and clarify the role of whey protein supplementation in preserving lean mass across different weight loss interventions, an area that remains underexplored compared with its well-established use in older adults in disease-related malnutrition. By integrating evidence from studies involving hypocaloric diets, bariatric surgery, and emerging pharmacological treatments, despite the absence of data for GLP-1 receptor agonists, this review seeks to highlight the potential clinical relevance of whey protein in modern weight loss strategies and to support the development of future research in this rapidly evolving therapeutic landscape.

The aim of this review is to assess the effectiveness of whey protein supplementation, compared with placebo or standard interventions, in preserving skeletal muscle mass and functional outcomes in adults with obesity undergoing weight loss treatments, including hypocaloric diets combined with pharmacological therapy or bariatric surgery.

## 2. Materials and Methods

### 2.1. Study Design

Our systematic review was executed and reported in accordance with the Preferred Reporting Items for Systematic Reviews and Meta-Analyses (PRISMA) statement [20]. The protocol was registered in PROSPERO with code CRD420251069996.

The review question posed was whether whey protein supplements are more effective than placebo or standard interventions in preserving fat-free mass and functional outcomes in adults with obesity undergoing weight loss treatments, including hypocaloric diets combined with pharmacological therapy or bariatric surgery.

The objectives of this review are to evaluate the effectiveness of whey protein supplementation in preserving fat-free mass in adults with obesity undergoing weight loss interventions, including hypocaloric diets, pharmacological treatments, or bariatric surgery, to assess its impact on physical function and muscle strength during these interventions, to compare body-composition outcomes between whey protein supplementation and placebo or standard care, and to examine the safety profile and adherence associated with whey protein use within multimodal obesity treatment strategies.

Across all outcomes, changes in fat-free mass were consistently reported using mean difference with 95% confidence intervals as the effect measure, regardless of the assessment method (DXA, BIA, MRI, or underwater weighing) or the specific comparison evaluated (whey protein, hydrolysate, milk protein isolate, fortified yogurt, essential amino acid meal replacements, or combined protein formulations versus placebo, carbohydrate controls, isocaloric diets, or standard care).

### 2.2. Search Strategy

We conducted a systematic search of several electronic databases, PubMed/MEDLINE, Cochrane Central Register of Controlled Trials (CENTRAL), Embase, Scopus, ClinicalTrials.gov and WHO International Clinical Trials Registry Platform (ICTRP), to identify relevant literature published in English. We selected Randomized Controlled Trials (RCTs). Our search strategy employed a combination of keywords and medical subject headings (MeSH) to ensure comprehensive coverage of the literature. Specifically, we used the following search terms: (“Whey Proteins” [Mesh] OR “whey protein” OR “whey protein supplement*” OR “milk protein”) AND (“Obesity” [Mesh] OR obesity OR “overweight”) AND (“Weight Loss” [Mesh] OR “weight loss” OR “body weight reduction” OR “fat loss”) AND (“Randomized Controlled Trial” [Publication Type] OR “randomized controlled trial” OR “RCT”) NOT (“Child” [Mesh] OR “Adolescent” [Mesh] OR pediatric OR “chronic kidney disease” OR “renal failure” OR “chronic liver disease” OR “liver cirrhosis” OR “cancer” OR “neoplasm” OR “oncology”).

This review includes randomized controlled trials (RCTs) involving adults over 18 years of age with obesity (BMI > 30 kg/m^2^). The intervention of interest is whey protein supplementation provided as part of a hypocaloric diet, compared with placebo, a standard diet, or non-protein supplements. Primary outcomes include changes in fat-free mass, assessed using validated techniques such as dual-energy X-ray absorptiometry (DXA), bioelectrical impedance analysis (BIA), or magnetic resonance imaging (MRI), as well as muscle-function measures evaluated through standardized methods. Secondary outcomes encompass changes in total body weight, fat mass, BMI, metabolic markers such as glucose and lipid levels, adherence to the intervention, and adverse effects. Studies were excluded if they involved participants under 18 years of age or individuals with chronic kidney disease, chronic liver disease, or active cancer.

### 2.3. Review Process

Two independent reviewers screened the titles and abstracts of all identified studies, followed by a full-text assessment, with any discrepancies resolved through consensus or consultation with a third reviewer. The methodological quality of the included studies was evaluated using the Cochrane Risk of Bias 2.0 tool [21]. To ensure certainty in the body of evidence, the GRADE Pro tool were applied [22], as GRADE assesses the certainty of evidence across studies for each outcome rather than the quality of individual studies. This framework classifies certainty into four levels: high, indicating very high confidence that the true effect is close to the estimate; moderate, reflecting reasonable confidence that the true effect is close to the estimate; low, reflecting limited confidence with a meaningful possibility that the true effect differs substantially; and very low, signifying minimal confidence in the effect estimate. The search, review, critical appraisal, and analysis of the articles were conducted between March 2025 and September 2025. Compliance with PRISMA 2020 checklist was ensured throughout the review process, and the completed checklist is provided as Supplementary S2.

## 3. Results

**Figure 1:**
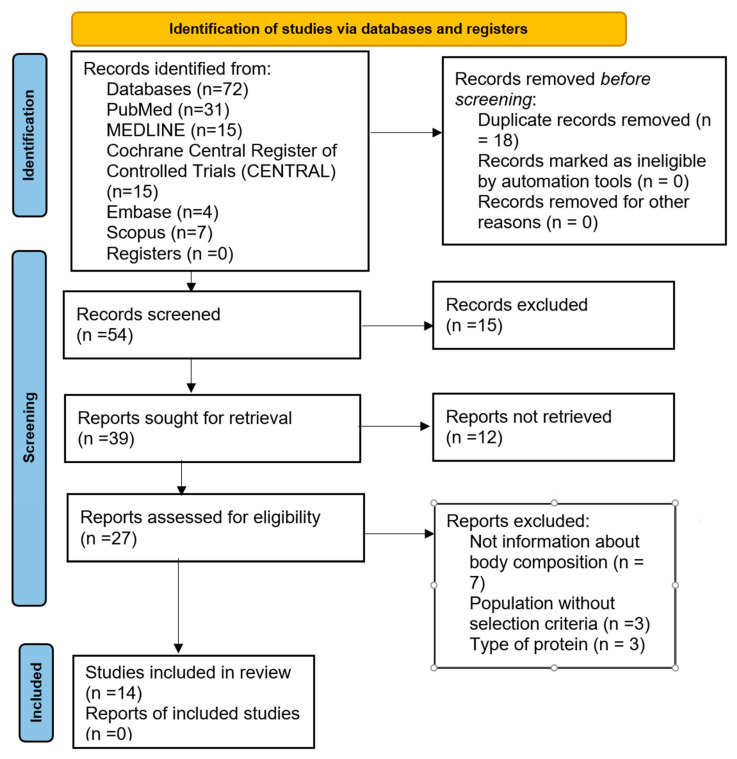
Flow diagram of the study selection process.

The search strategy yielded a total of 144 potential articles. After removing 18 duplicates and screening the records, we assessed 27 articles for eligibility. Seven articles were excluded for not reporting body composition outcomes, three due to sample-related exclusion criteria, and three based on the type of protein studied. Ultimately, a total of 14 RCTs were included in our review. The selection process in PRISMA 2020 Flow Diagram is summarized in Figure 1.

### 3.1. Study Population

A total of 14 clinical trials were included in the review. The study samples consisted of adults over 18 years of age with obesity. Only two trials enrolled patients who had previously undergone bariatric surgery, specifically Roux-en-Y gastric bypass [23,24]. Five studies included postmenopausal women [24,25,26,27,28]. Two studies enrolled participants with prediabetes or type 2 diabetes mellitus [28,29]. Female participants were predominant across all trials. The mean age ranged from 40 to 45 years, although two studies included individuals older than 80 years [25,30]. The mean BMI was 30–31 kg/m^2^, and one study included participants with BMI >40 kg/m^2^ [26].

### 3.2. Type of Protein

All studies used whey protein as the primary protein source. Four trials included leucine supplementation [13,26,29,30]. Mohammadi-Sartang et al. administered a fortified yogurt containing whey protein, calcium, vitamin D_3_, prebiotics, and probiotics [31]. Verreijen et al. enriched whey protein with vitamin D_3_, leucine, and essential amino acids [13]. Liu et al. compared whey protein with plant-based soy protein [28].

Protein doses ranged from 20 g/day [25] to 50 g/day [32]. Several studies calculated protein intake based on ideal body weight [22,24,26,33,34].

### 3.3. Intervention

The mean follow-up duration ranged from 2 weeks [27] to 6 months [26,28]. All studies included a control group and implemented a hypocaloric diet alongside protein supplementation. Hudson et al. [33], Kjølbæk et al. [35], and Claessens et al. [32] administered maltodextrin to the control group. Lamarca et al., Memelink et al., Ellegaard Larsen et al., and Verreijen et al. incorporated structured physical activity as part of the intervention [13,23,29,34].

### 3.4. Fat-Free Mass

In all studies, the assessment of changes in fat-free mass was included as either a primary or secondary outcome. Body composition was measured at baseline and at the end of the intervention. The methods used to assess body composition varied across studies. Sun et al., Lopes Gomes et al., Haidari et al., Mohammadi-Sartang et al., Coker et al., and Liu et al. employed bioelectrical impedance analysis [24,25,27,28,29,30]. Lamarca et al., Memelink et al., Hudson et al., Ellegaard Larsen et al., Verreijen et al., and Kjølbæk et al. used dual-energy X-ray absorptiometry (DXA), Gordon et al. used magnetic resonance imaging, and Claessens et al. used underwater weighing (hydrodensitometry) [13,23,29,32,33,34,35].

Sun et al., Lamarca et al., Memelink et al., Mohammadi-Sartang et al., and Verreijen et al. reported maintenance or increases in fat-free mass in the protein-supplemented groups compared with placebo, while the remaining studies found no significant differences [13,23,25,29,31].

Table 1 summarizes the characteristics and outcomes of each study with whey protein supplementation alone, and Table 2 summarizes the characteristics of studies with combined whey protein with exercise.

### 3.5. Risk of Bias Assessment

**Figure 2:**
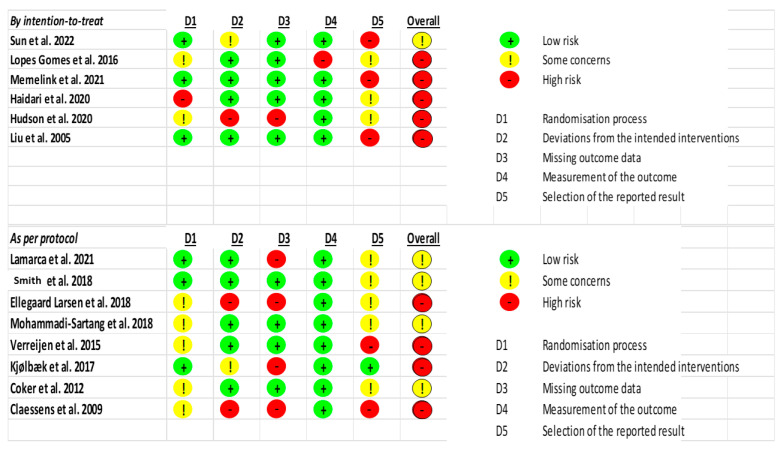
Cochrane Risk of Bias 2.0. [13,23,24,25,26,27,28,29,30,31,32,33,34,35].

The Cochrane Collaboration’s risk-of-bias tool, including the risk-of-bias summary and risk-of-bias graph, was used to evaluate methodological quality, as shown in Figure 2 and Supplementary S1. The most frequent sources of bias were small sample sizes, lack of blinding, wide confidence intervals, and limited effect estimates. Overall, the risk of bias was rated as moderate to high in most of the included studies.

Across both the intention-to-treat and per-protocol analyses, domain D2 (deviations from the intended interventions) consistently shows either high risk or some concerns, making it the most frequently problematic domain. This trend is evident in nearly all studies that did not receive a low-risk overall judgment. Additionally, D1 (Randomization process) emerges as another recurrent source of bias, particularly in the per-protocol group, where several studies [13,32,35] were rated as high-risk due to issues in randomization. Therefore, rather that relegating this insight to visual summaries, the authors should explicitly state in the main text that D2 and D1 are the most commonly flagged domains for bias, as this has direct implications for the internal validity and interpretability of the included studies.

### 3.6. Certainty of Evidence

Randomized trials evaluating whey protein and other protein-based interventions report small and inconsistent effects on fat-free mass, as assessed by DXA, BIA, MRI, and related methods. Reported mean differences range from slight reductions to modest gains, accompanied by wide confidence intervals and substantial imprecision. The certainty of the evidence, assessed with GRADE, varies from very low to moderate, largely due to factors such as small sample sizes and considerable heterogeneity. (Supplementary S1).

## 4. Discussion

The present systematic review provides a comprehensive synthesis of randomized controlled trials evaluating the effectiveness of whey protein supplementation in preserving fat-free mass (FFM) during weight loss interventions in adults with obesity. This topic is of increasing clinical relevance, as contemporary perspectives on obesity extend beyond excess adiposity to include alterations in skeletal muscle mass, muscle quality, and function, all of which have profound metabolic, endocrine, and functional implications [3,4]. Skeletal muscle is now recognized as a key regulator of glucose metabolism, inflammation, and whole-body energy homeostasis through the secretion of myokines, positioning muscle preservation as a central therapeutic goal in obesity management [3].

Weight loss, whether achieved through hypocaloric diets, pharmacological therapies, or bariatric surgery, is frequently accompanied by unintended reductions in FFM. This phenomenon is particularly concerning in populations already predisposed to muscle loss, such as older adults, postmenopausal women, individuals with type 2 diabetes, and patients undergoing rapid or substantial weight reduction after bariatric procedures [5,6,8]. The coexistence of excess fat mass and reduced skeletal muscle mass—defined as sarcopenic obesity—is associated with increased cardiovascular risk, impaired physical function, higher rates of falls and fractures, and increased mortality compared with obesity alone [4,7]. Consequently, strategies aimed at preserving fat-free mass during weight loss are essential to ensure not only metabolic improvement but also long-term functional health.

Across the 14 randomized controlled trials included in this review, whey protein supplementation was evaluated in diverse clinical contexts, reflecting the heterogeneity of obesity phenotypes encountered in real-world practice. Study populations varied widely in age, sex distribution, baseline metabolic status, and intervention type, ranging from short-term hypocaloric diets to long-term lifestyle interventions and post-bariatric surgery care. While this heterogeneity limits the ability to draw uniform conclusions, it also highlights important contextual factors that may modulate the effectiveness of protein supplementation.

One consistent observation is that whey protein supplementation appears to be more effective in preserving FFM when implemented as part of a multimodal intervention that includes structured physical activity, particularly resistance training [12,13]. Muscle protein synthesis is known to be synergistically stimulated by dietary amino acids and mechanical loading, and the absence of either component may blunt the anabolic response [5,12]. In this regard, studies such as those by Lamarca et al. and Memelink et al. demonstrated significant improvements in FFM when whey protein supplementation was combined with resistance exercise, whereas supplementation alone often resulted in neutral effects [23,29]. These findings reinforce current recommendations that nutritional and exercise interventions should be integrated rather than applied in isolation in obesity treatment [5].

Age emerged as another critical determinant of response. Trials conducted in older adults, including postmenopausal women and individuals aged over 55 years, tended to report more favorable effects of whey protein supplementation on FFM preservation [12,13]. Aging muscle exhibits anabolic resistance, characterized by a diminished sensitivity to dietary protein and insulin-mediated anabolic signals [9,12]. Whey protein, due to its high biological value, rapid digestibility, and rich leucine content, may partially overcome this resistance by providing a strong stimulus for muscle protein synthesis. This effect may be further enhanced when whey protein is enriched with leucine, essential amino acids, or vitamin D, as observed in several included studies [13,18]. In fact, four of the randomized controlled trials incorporated leucine-enriched formulations, highlighting its relevance as a key anabolic component [13,26,29,30]. These studies underscore that leucine plays a central mechanistic role in overcoming anabolic resistance in older adults, particularly when combined with resistance exercise [13,26,29,30]. However, despite these references, most studies did not report participants’ overall dietary protein intake, limiting the ability to determine whether the observed benefits reflect compensation for a pre-existing protein deficit or the intrinsic anabolic properties of leucine-containing formulations.

The role of protein dose and formulation also warrants consideration. Protein supplementation regimens varied substantially across trials, with doses ranging from 20 g/day to 50 g/day or calculated relative to ideal body weight. This variability largely reflects differences in study aims, population characteristics, and dietary backgrounds. Some trials used absolute daily doses, whereas others individualized supplementation according to body weight to better account for metabolic needs, particularly in patients with obesity or those undergoing rapid weight loss [5,7]. Studies employing higher protein intakes or individualized dosing strategies tended to demonstrate more consistent preservation of FFM, particularly in settings of energy restriction or rapid weight loss. These findings align with evidence suggesting that standard protein recommendations may be insufficient during weight loss, especially in older or metabolically compromised individuals [11,12,14]. Nevertheless, the lack of standardized dosing protocols across studies remains a significant limitation and underscores the need for future trials to directly compare different protein doses and timing strategies.

Bariatric surgery represents a unique clinical scenario in which the risk of fat-free mass loss is particularly pronounced. Rapid postoperative weight loss, reduced food intake, altered digestion, and nutrient malabsorption can all contribute to accelerated muscle catabolism if nutritional intake is not adequately managed [6,15,16]. In this review, studies involving post-bariatric patients yielded mixed results. Lamarca et al. reported significant gains in FFM with combined protein supplementation and resistance training [23], whereas Lopes Gomes et al. observed no statistically significant differences between higher and standard protein intake groups [24]. These discrepancies may be attributable to differences in study design, duration, assessment methods, and the presence or absence of structured exercise programs. Importantly, these findings suggest that protein supplementation alone may be insufficient to counteract muscle loss in post-bariatric patients without concurrent mechanical stimulation and comprehensive nutritional support.

Methodological variability across studies represents another major factor influencing outcomes. Body composition assessment techniques included bioelectrical impedance analysis (BIA), dual-energy X-ray absorptiometry (DXA), magnetic resonance imaging (MRI), and underwater weighing. While BIA is widely used due to its practicality, it is sensitive to hydration status and may be less accurate during periods of rapid weight change [3,9]. In contrast, DXA and MRI provide more precise and reproducible measurements of fat-free mass and its distribution, which may explain why studies using these techniques more frequently detected modest but significant differences in FFM. Given these differences, it is important to distinguish findings derived from DXA/MRI-based studies, which offer higher measurement validity. MRI allows detailed evaluation of abdominal adipose tissue and liver fat [36], while DXA can quantify total and regional fat mass as well as visceral adipose tissue, capturing metabolically relevant changes in fat redistribution [37]. In contrast, studies relying on BIA are more susceptible to error, as fluctuations in hydration and fluid shifts during weight loss may lead to misestimation of fat-free mass, including in patients with varying protein intakes during staged diet progression after sleeve gastrectomy [38]. When interpreting BIA, hydration status and cellular components should be carefully considered during weight loss to correctly characterize changes in these compartments [39]. This methodological heterogeneity likely contributed to the inconsistency observed across trials and is reflected in the GRADE assessments, where certainty of evidence ranged from very low to moderate depending on the outcome and measurement method.

Several trials included in this review reported neutral findings, with no significant differences in FFM preservation between whey protein supplementation and control interventions [17]. These results should be interpreted cautiously. In many cases, neutral findings occurred in studies with short follow-up durations, small sample sizes, or insufficient protein doses relative to the degree of energy restriction. For example, very-low-calorie diet interventions may induce a catabolic state that overwhelms the anabolic stimulus provided by protein supplementation alone, particularly in the absence of resistance exercise [5,14]. Additionally, studies comparing whey protein with other high-quality protein sources, such as soy or casein, often found comparable effects on FFM, suggesting that overall protein adequacy may be more important than protein source in certain contexts [17].

Beyond changes in body composition, the preservation of muscle function and strength is a clinically meaningful outcome that was not uniformly assessed across studies. Muscle mass and muscle function do not always change in parallel, and functional measures such as strength, power, and physical performance are more directly linked to quality of life, independence, and long-term health outcomes [7,8]. Trials that incorporated resistance training and assessed functional outcomes tended to support the notion that maintaining FFM may translate into preserved or improved physical function. This is particularly relevant for older adults and post-bariatric patients, in whom functional decline can have substantial clinical consequences. The isolated effect of whey protein on muscle mass and function has not been conclusively demonstrated. However, when combined with resistance training, whey protein intake appears to produce greater improvements than resistance training alone in individuals with sarcopenia [40] and sarcopenic obesity [41].

From a metabolic perspective, preserving FFM during weight loss may also facilitate better long-term weight maintenance. Skeletal muscle is a major determinant of resting energy expenditure, and excessive loss of fat-free mass can reduce energy requirements, potentially predisposing individuals to weight regain [10,11]. Moreover, muscle tissue plays a central role in glucose disposal and insulin sensitivity, linking muscle preservation to improved glycemic control and reduced cardiometabolic risk [8]. In this context, whey protein supplementation may offer indirect metabolic benefits beyond its effects on body composition, although these outcomes were not consistently reported across included trials.

A key limitation of this review is the limited availability of studies evaluating isolated whey protein in patients experiencing weight loss secondary to therapeutic interventions, such as pharmacological treatments or bariatric surgery. Because of this, several of the included studies assessed whey protein as part of multi-ingredient supplements, which makes it difficult to attribute observed effects exclusively to whey protein. Although isolated whey protein has been more investigated in primary sarcopenia, evidence within the specific clinical context of weight loss-associated muscle deterioration remains insufficient, restricting the ability to get conclusions about its isolated impact. Another limitation of this review is that most included studies did not provide detailed information on participants’ baseline protein intake or on potential changes in habitual dietary protein consumption during the intervention. Although the sources indicate that an initial nutritional assessment and baseline and post-intervention body compositions were performed, the generally do not specify whether whey supplementation compensated for a pre-existing protein deficit or exerted and intrinsic anabolic effect. It is broadly recognized that protein deficiency is common among individuals with obesity, whose dietary patterns prior to intervention often emphasize carbohydrates and saturated fats. This lack of precise reporting restricts our ability to determine the specific contribution of whey protein and requires cautious interpretation of its isolated impact. Another important limitation is the small sample sizes across studies, which reduce statistical power and limit the robustness of the findings, while the marked sex imbalance, characterized by a predominance of female participants, restricts the generalizability of the results to broader populations. Additionally, the short follow-up durations commonly reported in the trials hinder the evaluation of long-term effects. The possibility of publication bias must also be considered, given the limited number of studies and the likelihood that positive results are more frequently disseminated. Lastly, this review focused on body composition outcomes; however, whey protein and/or exercise may also influence functional parameters such as muscle strength, which were not assessed or included in our search strategy. Future studies should specifically address these functional components.

The overall certainty of evidence, as evaluated using the GRADE framework, was limited by recurrent methodological issues, including small sample sizes, wide confidence intervals, heterogeneity of interventions, and moderate-to-high risk of bias in several studies. The included trials differed substantially in protein dose, type of formulation, duration of the intervention, and participant characteristics, all of which contributed to marked heterogeneity and reduced comparability across studies. In addition, the use of different body-composition assessment methods (BIA, DXA, or MRI) further limited consistency in outcome measurement. Many trials also presented methodological weaknesses such as lack of blinding imprecise estimates, which increased the risk of bias. The predominance of female participants restricts the generalizability of the findings to broader populations. Moreover, because the available evidence did not allow for a meta-analysis, the review relied on qualitative synthesis, resulting in a certainty of evidence that is mostly low to moderate and conclusions that remain primarily descriptive rather than quantitative.

Despite these limitations, the balance of evidence suggests that whey protein supplementation can contribute to the preservation of fat-free mass during weight loss in adults with obesity, particularly when implemented as part of a comprehensive, multimodal intervention that includes resistance exercise and adequate total protein intake [12,13,14].

Future research should focus on well-powered randomized controlled trials with standardized protein dosing strategies, clearly defined exercise protocols, longer follow-up periods, and robust body composition and functional assessments. Identifying subgroups most likely to benefit from supplementation—such as older adults, individuals with diabetes, or post-bariatric patients—will be essential to refine clinical recommendations and advance precision nutrition approaches in obesity management [10,12]. Additionally, greater emphasis should be placed on clinically meaningful outcomes, including physical function, quality of life, and long-term weight maintenance, rather than body composition alone.

## 5. Conclusions

In conclusion, whey protein supplementation appears to support the preservation of fat-free mass during weight loss in adults with obesity, particularly when combined with resistance exercise and anabolic-enriched formulations. The benefits are more evident in older adults, metabolically compromised individuals, and post-bariatric surgery patients, whereas supplementation alone often yields neutral effects. Considerable heterogeneity in study design, protein dosing, and assessment methods limits definitive conclusions. Nonetheless, the available evidence underscores the importance of integrating adequate protein intake into multimodal obesity treatment strategies aimed at achieving high-quality weight loss and preventing the development or progression of sarcopenic obesity.

## Figures and Tables

**Table 1:** Summary of the characteristics and outcomes of studies with whey supplementation alone.

Study	Type of Protein	Tracking Time	Sample	Groups	Fat-Free Mass Determination Technique	Main Results on Free Fat Mass
Sun et al. 2022 [25]	Whey protein ad whey protein hydrolysate	8 weeks	Women aged 50–80 years Overweight or obese (BMI 24–35 kg/m^2^)	*Control:* hypocaloric diet (−500 kcal/day) *WP:* hypocaloric diet + 20 g/d whey protein *WPH:* hypocaloric diet + 20 g/d whey protein hydrolysate	InBody S10 (Biospace Co., Ltd., Seúl, Corea)	Control: –0.41 ± 1.04 WP: –0.03 ± 1.11 WPH: +0.11 ± 0.84
Smith et al. 2018 [26]	Whey protein isolate, leucine	6 months	70 postmenopausal women, 50–65 years old All with obesity (BMI 30–50 kg/m^2^)	*WM:* hypocaloric diet *WL:* hypocaloric diet + 0.8 g/Kg/day WL-PS: hypocaloric diet + 1.2 g/Kg/day	Nuclear Magnetic Resonance	WM: −0.14 WL: −1.80 WL-PS: −1.30
Lopes Gomes et al. 2017 [24]	Whey protein	16 weeks	30 women Age: 45 ± 11 years BMI: 35.7 ± 5.2 kg/m^2^ All had regained ≥5% of their lowest postoperative weight All underwent Roux-en-Y gastric bypass	*Control:* hypocaloric diet + 1 g/kg ideal body weight/day protein *Intervention group:* hypocaloric diet *+* Whey protein 1.5 g/kg ideal body weight/day	RJL Systems—Quantum BIA-101Q	Control group: −0.6 kg vs. Intervention group: 1.1 kg; *p* = 0.188
Haidari et al. 2020 [27]	Whey protein	2 weeks	60 premenopausal women Age: 31.6 ± 5.7 years BMI: 33.45 ± 2.89 kg/m^2^ All with obesity (BMI 30–40)	*Control group:* hypocaloric diet (−800 kcal/day) *Intervention group:* hypocaloric diet (−800 kcal/day) + 30 g/day whey protein	TANITA BC-418	Intervention: −1.00% (CI −1.38 to −0.63) Control: −2.02% (CI −2.36 to −1.69)
Hudson et al. 2020 [33]	Milk protein isolate (MPI)	20 weeks	44 adults with overweight or obesity Age: 52 ± 1 years BMI: 31.4 ± 0.5 kg/m^2^	*Control group:* hypocaloric diet + 0.7 g/kg/day carbohydrate (maltodextrin) *MPI:* hypocaloric diet + 0.7 g/kg/day milk protein isolate	Dual absorptiometry of X-Ray (DXA)	Control group: –1.1 ± 0.2 kg MPI: –1.3 ± 0.3 *p* > 0.05
Mohammadi-Sartang et al. 2018 [31]	Fortified yogurt (FY) containing: Whey protein: 5 g per serving (10 g/day) Calcium: 500 mg per serving Vitamin D3: 500 IU per serving Inulin (prebiotic): 3 g per serving Probiotic: Bifidobacterium lactis Bb-12 (≥10^7^ cfu/g)	10 weeks	87 participants completed the study Age: 20–65 years, mean ~45 BMI: 25–34.9 kg/m^2^ All diagnosed with metabolic syndrome	*Plain Yogurt:* Low-fat plain yogurt (2 × 250 g/day) + calorie-restricted diet *Fortified yogurt*: Fortified yogurt with whey protein, calcium, vitamin D, inulin, probiotics (2 × 250 g/day) + calorie-restricted diet	InBody S10 (Korea)	PY: –2.0 ± 2.7 kg FY: –0.9 ± 3.5 kg *p* = 0.025
Kjølbæk et al. 2017 [35]	Whey + calcium (whey+): 45 g/day Whey protein (highα-lactalbumin) 1000 mg/day calcium Whey: 45 g/day whey protein Soy: 45 g/day soy protein isolate	32 weeks	151 adults, aged 18–60 years, BMI 27.6–40.4 kg/m^2^	*Control group:* 48 g/day maltodextrin *Whey + calcium (whey+):* 45 g/day whey + 1000 mg Ca *Whey:* 45 g/day whey *Soy:* 45 g/day soy protein All with hypocaloric diet	DXA (Dual-energy X-ray Absorptiometry)	Control group: +1.74 ± 1.4 kg Whey + calcium (whey+): +1.87 ± 1.7 kg Whey: +1.94 ± 1.3 kg Soy: +1.58 ± 1.4 kg *p* = 0.50
Coker et al. 2012 [30]	EAAMR (Essential Amino Acid Meal Replacement): 7 g whey protein, 6 g added essential amino acids (40% leucine) CMR (Competitive Meal Replacement): 14 g caseinate protein	8 weeks	11 obese older adults (65–80 y) BMI ± 31	*EAAMR*: Meal replacement con whey + EAA *CMR*: Meal replacement with casein (control)	Bioelectrical impedance (BIA)	EAAMR: –2.1 ± 0.5 kg CMR: –3.6 ± 0.5 kg *p* = 0.26
Liu et al. 2005 [28]	Soy: 15 g/day soy protein + 100 mg isoflavones. Iso: 15 g/day milk protein + 100 mg isoflavones. Placebo: 15 g/day milk protein, no isoflavones	6 months	180 Chinese postmenopausal women. Age: 48–70 years. Condition: prediabetes or early untreated diabetes.	*Soy:* soy protein + isoflavones. *Iso*: milk protein + isoflavones. *Placebo*: milk protein only.	Tanita TBF-410-GS	FFM remained unchanged in all groups.
Claessens et al. 2009 [32]	Whey protein	18 weeks	48 obese adults (31 women, 17 men) Age: 30–60 years	*HC* (*High-Carbohydrate*): low-fat diet + maltodextrin (50 g/day) *HPC (High-Protein Casein):* low-fat diet + casein (50 g/day) *HPW (High-Protein Whey)*: low-fat diet + whey protein (50 g/day)	Underwater weighing (hydrodensitometry)	Total, sample: 56.8 → 55.0 kg (−1.8 kg) No significant differences between HC vs. HP or between casein vs. whey.

BMI: Body Mass Index; WP: Whey Protein; WPH: Whey Protein Hydrolysate; MPI: Milk Protein Isolate; FY: Fortified Yogurt; PY: Plain Yogurt; EAAMR: Essential Amino Acid Meal Replacement; CMR: Competitive Meal Replacement; FFM: Fat Free Mass; DXA: Dual-Energy X Absorptiometry; BIA: Bioelectrical Impedance Analysis.

**Table 2:** Summary of the characteristics and outcomes of studies with whey supplementation and exercise program.

Study	Type of Protein	Tracking Time	Sample	Groups	Fat-Free Mass Determination Technique	Main Results on Free Fat Mass
Lamarca et al. 2021 [23]	Whey protein	12 weeks	Adults 2–7 years after Roux-en-Y gastric bypass Women (≈90%) 40–45 years Post-surgical BMI: ~30 kg/m^2^	*Control:* no strength training, no protein supplementation *RT:* strength training *PRO:* 0.5 kg/day ideal body weight protein supplementation *RT + PRO:* strength training + protein supplementation	Dual-energy X-ray Absorptiometry	FFM: RTP + PRO +1.46 kg vs. control −0.24 kg *p* = 0.006 SMM: RTP + PRO +0.91 kg vs. control −0.08 kg; *p* = 0.008
Memelink et al. 2020 [29]	Whey protein, leucine and vitamin D3	13 weeks	123 older adults (≥55 years) Obesity + type 2 diabetes or prediabetes Age: 66–67 years BMI: 33 kg/m^2^ 65% men	*Control:* isocaloric non-protein drink+ hypocaloric diet + exercise program *Test group:* enriched whey protein drink+ hypocaloric diet + exercise program	Dual-energy X-ray Absorptiometry	Control −0.35 ± 0.26 vs. test group +0.57 ± 0.27; +0.92 kg (95% CI 0.19 to 1.65); *p* = 0.015
Larsen et al. 2018 [34]	Whey protein isolate	4 weeks	29 overweight or obese adults (BMI > 28 kg/m^2^) Age range: 21–55 years, mean ~41	*Control group*: VLCD (~690 kcal/day) + walking program *PRO:* VLCD + walking + bedtime whey protein isolate (0.4 g/kg/day)	Dual-energy X-ray absorptiometry (DXA)	Control group: –2.4 [–3.2; –1.6] PRO: –2.7 [–3.6; –1.7] *p* = 0.65
Verreijen et al. 2015 [13]	Whey protein: 21 g per serving Added leucine: 2.8 g Essential amino acids: 10.6 g Vitamin D3: 20 µg (800 IU)	13 weeks	60 obese older adults (≥55 y) Age: 63 ± 6 y BMI: 33 ± 4.4 kg/m^2^	*Intervention*: Whey + leucine + vitamin D supplement + hypocaloric diet + resistance training *Control:* Isocaloric control product + hypocaloric diet + resistance training	DXA (Dual-energy X-ray Absorptiometry)	Intervention –0.5 ± 2.1 kg Control –0.5 ± 2.1 kg β = +0.95 kg (*p* = 0.03)

BMI: Body Mass Index; RT: Resistance Training; PRO: Protein Supplementation; VLCD: Very Low Calorie Diet; FFM: Fat Free Mass; SMM: Skeletal Muscle Mass; DXA: Dual-Energy X Absortiometry.

## Data Availability

Not applicable.

## Supplementary Materials

The following supporting information can be downloaded at: https://www.mdpi.com/article/10.3390/nu18040695/s1, Supplementary S1: Summary of randomized trials assessing the effect of whey protein and other protein interventions on free fat mass. Data include mean difference (95% CI), number of participants, and certainty of evidence (GRADE). Supplementary S2: PRISMA 2020 Checklist.
