# Supplementary material for: Effectiveness of Whey Protein Supplementation in Weight Loss Interventions for Patients with Obesity: A Systematic Review
Source: Nutrients. 2026 Feb 21;18(4):695. doi: 10.3390/nu18040695 (PMC12942925; doi:10.3390/nu18040695)
Supplement: Supplementary file 1 [file nutrients-18-00695-s001.zip › Supplementary 1.pdf]

Certainty assessment							№ of patients		Effect		Certainty	Importance
№ of studies	Study design	Risk of bias	Inconsistency	Indirectness	Imprecision	Other considerations	Whey Protein	Standard Care	Relative (95% CI)	Absolute (95% CI)		
**Free fat mass (Whey protein VS placebo VS resistence training) (follow-up: mean 12 weeks; assessed with: DXA; Scale from: 0 to 10) [23]**												
1	randomised trials	serious[a]	serious[a]	not serious[b]	serious[b]	none	20	40	-	MD **1.1 Kg higher** (0.2 higher to 2 higher)	⊕◯◯◯ Very low[a,b]	IMPORTANT
**Free fat mass (whey protein VS placebo) (assessed with: NMR; Scale from: 0 to 50) [26]**												
1	randomised trials	not serious	serious[c]	not serious	serious[b]	none	24	46	-	MD **0.8 Kg higher** (0.3 lower to 0.3 higher)	⊕⊕◯◯ Low[b,c]	IMPORTANT
**Free fat mass (Whey protein VS control) (assessed with: BIA; Scale from: 0 to 50) [24]**												
1	randomised trials	serious[d]	serious[e]	not serious	serious[e]	none	15	15	-	MD **0 Kg** (0.5 lower to 0.5 higher)	⊕◯◯◯ Very low[d,e]	NO IMPORTANT
**Free fat mass (whey protein VS placebo) (assessed with: DXA; Scale from: 0 to 1) [29]**												
1	randomised trials	not serious	not serious	not serious	serious[a]	none	61	62	-	MD **0.28 Kg higher** (0.19 higher to 1.65 higher)	⊕⊕⊕◯ Moderate[a]	IMPORTANT
**Free fat mass (hipocaloric diet VS hipocaloric diet+wehy protein) (follow-up: mean 2 weeks; assessed with: BIA ; Scale from: 0 to 30) [25]**												

Certainty assessment							№ of patients		Effect		Certainty	Importance
№ of studies	Study design	Risk of bias	Inconsistency	Indirectness	Imprecision	Other considerations	Whey Protein	Standard Care	Relative (95% CI)	Absolute (95% CI)		
1	randomised trials	not serious	serious[c]	not serious	serious[a]	none	30	30	-	MD **0 1.02** (1 lower to 2.02 lower)	⊕⊕◯◯ Low[a,c]	IMPORTANT
**Free fat mass (Milk Protein Isolate VS Carbohidrato, maltodextrina) (assessed with: DXA; Scale from: 0 to 61) [33]**												
1	randomised trials	not serious	not serious	not serious	serious[f]	none	21	23	-	MD **0.2 lower** (1.1 lower to 1.3 lower)	⊕⊕⊕◯ Moderate[f]	IMPORTANT
**Free fat mass (VLCD VS VLCD+whey protein) (assessed with: DXA; Scale from: 0 to 50) [34]**												
1	randomised trials	not serious	not serious	not serious	serious[a]	none	14	15	-	MD **0.3 lower** (2.4 lower to 2.7 lower)	⊕⊕⊕◯ Moderate[a]	IMPORTANT
**Free fat mass (Fortified Yogurt VS standar yogurt) (assessed with: BIA ; Scale from: 0 to 55) [31]**												
1	randomised trials	not serious	not serious	not serious	serious[b]	none	44	43	-	MD **1.1 higher** (0.9 lower to 2 lower)	⊕⊕⊕◯ Moderate[b]	IMPORTANT
**Free fat mass (whey protein VS isocaloric diet) (assessed with: DXA; Scale from: 0 to 2) [13]**												

Certainty assessment							№ of patients		Effect		Certainty	Importance
№ of studies	Study design	Risk of bias	Inconsistency	Indirectness	Imprecision	Other considerations	Whey Protein	Standard Care	Relative (95% CI)	Absolute (95% CI)		
1	randomised trials	not serious	not serious	not serious	very serious[f]	none	30	30	-	MD **0.95 Kg higher** (0.09 higher to 1.81 higher)	⨁⨁◯◯ Low[f]	NO IMPORTANT
**Free fat mass (whey + calcio, whey, soja, control) (assessed with: DXA; Scale from: 0 to 2) [35]**												
1	randomised trials	not serious	not serious	not serious	serious[b]	none	77	74	-	MD **0.06 Kg higher** (0.47 lower to 0.59 higher)	⨁⨁⨁◯ Moderate[b]	IMPORTANT
**Free fat mass (Essential Amino Acid Meal Replacement VS Competitive Meal Replacement) (assessed with: BIA; Scale from: 0 to 3) [30]**												
1	randomised trials	not serious	not serious	not serious	serious[b]	none	6	5	-	MD **0.8 Kg higher** (0.7 lower to 2.3 higher)	⨁⨁⨁◯ Moderate[b]	IMPORTANT
**Free fat mass (isoflavonas+whey proteina VS placebo) (assessed with: BIA; Scale from: 0 to 1) [28]**												
1	randomised trials	not serious	not serious	not serious	serious[f]	none	120	60	-	MD **0.65 Kg lower** (2 lower to 0.7 higher)	⨁⨁⨁◯ Moderate[f]	IMPORTANT
**Free fat mass (whey protein VS protein casein VS carbohydrates) (assessed with: underwater weighing) [32]**												

Certainty assessment							№ of patients		Effect		Certainty	Importance
№ of studies	Study design	Risk of bias	Inconsistency	Indirectness	Imprecision	Other considerations	Whey Protein	Standard Care	Relative (95% CI)	Absolute (95% CI)		
1	randomised trials	not serious	not serious	not serious	serious[b,f]	none	32	16	-	MD **0.08 Kg lower** (0.9 lower to 0.7 higher)	⨁⨁⨁◯ Moderate[b,f]	IMPORTANT
**Free fat mass (whey protein hydrolysate VS placebo VS whey protein) (assessed with: BIA; Scale from: 0 to 38) [25]**												
1	randomised trials	serious[a]	not serious	not serious	serious[a]	none	40	20	-	MD **0.6 Kg higher** (0.5 lower to 0.3 higher)	⨁⨁◯◯ Low[a]	IMPORTANT

CI: confidence interval; **MD:** mean difference

Explanations

a. Limited sample size

b. Wide confidence interval

c. Heterogeneity

d. Study only with older female patients, without ensuring adherence to supplementation

e. Open-label design, absence of a placebo group

f. Small effect size

Supplementary 1. Summary of randomized trials assessing the effect of whey protein and other protein interventions on free fat mass. Data include mean difference (95% CI), number of participants, and certainty of evidence (GRADE).
